# Gene network inherent in genomic big data improves the accuracy of prognostic prediction for cancer patients

**DOI:** 10.18632/oncotarget.20548

**Published:** 2017-08-24

**Authors:** Yun Hak Kim, Dae Cheon Jeong, Kyoungjune Pak, Tae Sik Goh, Chi-Seung Lee, Myoung-Eun Han, Ji-Young Kim, Liu Liangwen, Chi Dae Kim, Jeon Yeob Jang, Wonjae Cha, Sae-Ock Oh

**Affiliations:** ^1^ Department of Anatomy, School of medicine, Pusan National University, Yangsan, 50612, Republic of Korea; ^2^ Department of Statistics, Korea University, Seoul 02841, Republic of Korea; ^3^ Department of Nuclear Medicine, Pusan National University Hospital, Busan 49241, Republic of Korea; ^4^ Department of Orthopaedic Surgery, Pusan National University Hospital, Busan 49241, Republic of Korea; ^5^ Biomedical Research Institute, Pusan National University Hospital and School of Medicine, Pusan National University, Busan 49241, Republic of Korea; ^6^ Department of Pharmacology, School of medicine, Pusan National University, Yangsan, 50612, Republic of Korea; ^7^ BEER, Busan society of Evidence-based mEdicine and Research, Busan 49241, Republic of Korea; ^8^ Department of Otorhinolaryngology-Head and Neck Surgery, Pusan National Hospital, Busan 49241, Republic of Korea

**Keywords:** prognosis, network-regularized high-dimensional Cox-regression (Net), breast cancer, gene network, gene signature

## Abstract

Accurate prediction of prognosis is critical for therapeutic decisions regarding cancer patients. Many previously developed prognostic scoring systems have limitations in reflecting recent progress in the field of cancer biology such as microarray, next-generation sequencing, and signaling pathways. To develop a new prognostic scoring system for cancer patients, we used mRNA expression and clinical data in various independent breast cancer cohorts (n=1214) from the Molecular Taxonomy of Breast Cancer International Consortium (METABRIC) and Gene Expression Omnibus (GEO). A new prognostic score that reflects gene network inherent in genomic big data was calculated using Network-Regularized high-dimensional Cox-regression (Net-score). We compared its discriminatory power with those of two previously used statistical methods: stepwise variable selection via univariate Cox regression (Uni-score) and Cox regression via Elastic net (Enet-score). The Net scoring system showed better discriminatory power in prediction of disease-specific survival (DSS) than other statistical methods (p=0 in METABRIC training cohort, p=0.000331, 4.58e-06 in two METABRIC validation cohorts) when accuracy was examined by log-rank test. Notably, comparison of C-index and AUC values in receiver operating characteristic analysis at 5 years showed fewer differences between training and validation cohorts with the Net scoring system than other statistical methods, suggesting minimal overfitting. The Net-based scoring system also successfully predicted prognosis in various independent GEO cohorts with high discriminatory power. In conclusion, the Net-based scoring system showed better discriminative power than previous statistical methods in prognostic prediction for breast cancer patients. This new system will mark a new era in prognosis prediction for cancer patients.

## INTRODUCTION

**Figure 1:**
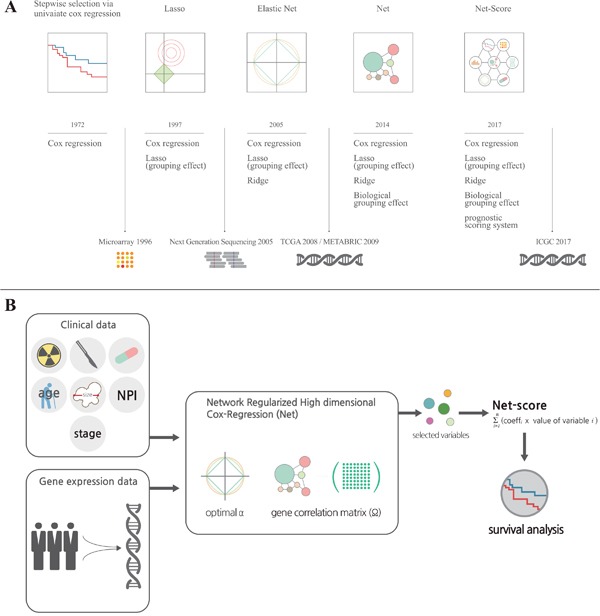
Development of statistical methods for prognostic prediction of cancer patients **(A)** It shows a brief history of the development of statistical methods for variable selection on a timeline. For more details, please refer to the description in “Results”. **(B)** It is a graphical summary for development of the Net-based scoring system. Total variables including clinical and genetic factors were assessed in Network Regularized High dimensional Cox-Regression (Net) with optimal parameters (gene expression correlation: α and gene network correlation: Ω). After variable selection, we generated a Net-score using estimated regression coefficient and variables ∑(coefficient X value of variable) from Net results.

Accurate prediction of prognosis is critical for therapeutic decisions in cancer patients. There is a long history of trials for accurate prognosis prediction, dating back to 1958 [1, 2]. Early statistical methods for survival analysis (life table or Kaplan-Meier method) were univariate analyses [1, 2]. To incorporate various types of clinical information on patients into the survival analysis, Cox proposed a multivariate proportional hazard model in 1972 (Figure 1A) [1]. Recent substantial advances in biomedical technology, such as microarray techniques and next-generation sequencing, have provided the possibility for better prognostic prediction [3, 4]. Although genome-wide information from cancer tissues has been accumulating worldwide, the statistical methods to incorporate this information into survival analyses are still not satisfactory to fulfill the medical need.

Microarrays or next-generation sequencing provides many variables (number of genes, large dimensionality) relative to the number of patients examined (sample size). To enhance the accuracy and efficiency of analysis from such tremendous databases, it is necessary to select and shrink variables in a systemic statistical manner in terms of the principle of parsimony. For this purpose, the least absolute shrinkage and selection operator (Lasso, 1997) (Figure 1A) and Ridge regression methods have been introduced [2, 5]. These methods provided a smart solution for the multicollinearity problem by adding a degree of bias to the regression estimates. Moreover, the Elastic Net regression method combines Lasso and Ridge methods to use advantages of both methods and provide a better group effect from correlation [5–7]. However, Lasso, Ridge, and even Elastic Net methods do not reflect recent progresses in cancer biology even though they reflect expressional changes of genes. To overcome this limitation, a novel statistical method (Network-Regularized high-dimensional Cox-regression, or Net) was recently developed [5]. Although Net reflects recent progress in cancer biology such as signaling pathways and network of genes, it has not yet been tested using big databases.

To derive accurate prognostic prediction, an appropriate data set is mandatory. With progress in biomedical technology, several big genomic databases of good quality have been released, such as The Cancer Genome Atlas (TCGA), Molecular Taxonomy of Breast Cancer International Consortium (METABRIC) and Gene Expression Omnibus (GEO). In addition, the International Cancer Genome Consortium (ICGC) will open in 2017. This provides a good opportunity to apply a new statistical method for prognostic prediction. One of the most important criteria to confirm the reliability of a new statistical method is a thorough external validation, which requires carefully collected independent data sets. These data sets should have the same clinical information and genomic data collected by the same experimental tool, as well as a large number of patients (>100). For accurate external validation, the following conditions must be met: (1) totally different cohorts, with at least one from a training cohort; (2) genomic data collected by the same technique because the absolute expression value of a gene can be different according to experimental methods such as microarray, RNAseq, and RT-PCR; (3) the cutoff value of risk groups used in the training cohort should also be applied in validation cohorts. In the current study, we used luminal breast cancer microarray data from METABRIC that satisfies the above conditions and consists of three independent cohorts. For the further validation of its versatility, we also applied it to other breast cancer cohorts (GSE7390, GSE42568, GSE22219 and GSE37181) from Gene Expression Omnibus (GEO) [8–14].

## RESULTS

### Variable selection

For comparison of the discriminatory accuracy of statistical methods, we chose five independent cohorts from the METABRIC database as described in “Methods”. Among five cohorts, we could not help excluding two cohorts because patients in two cohorts did not have clinical information like tumor stage and surgery types. After that, we used the cohort with the largest number of patients as a training set. To choose the best combination of variables including all examined genes, we applied three kinds of statistical method for disease-specific survival (DSS) as described in “Methods”: network-regularized high-dimensional Cox-regression (Net), stepwise selection via univariate Cox proportional hazard model, or Elastic net. At the process of variable selection, we included almost all clinical variables such as age, stage, Nottingham prognostic index (NPI), therapeutic modalities, hormonal status and tumor size. Among clinical variables, tumor size was always selected at the variable selection whether we changes the values of parameters or not. We identified 82, 47, and 102 variables, including tumor size, respectively, for each statistical method. Next, we generated a scoring system based on regression coefficients and variables (∑(coefficient X value of variable)). The regression coefficients and name of variables scored by three models are listed in Supplementary Table 1. We named the prognostic score of each statistical method the Net-score, Uni-score, and Enet-score. Simple explanation of the Net-based scoring system are described in Figure 1B and “Method”.

### Survival analysis

**Figure 2:**
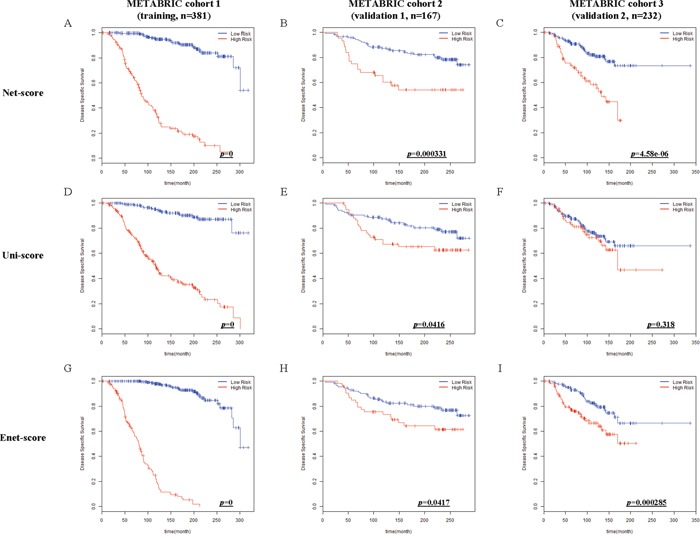
Kaplan-Meier estimates of survival of breast cancer patients according to the risk scores (Net-score, Uni-score, and Enet-score) Disease-specific survival (DSS) in METABRIC cohort 1 (training), METABRIC cohort 2 and 3 (validation 1 and 2) were examined by Net-score **(A, B** and **C)**, Uni-score **(D, E** and **F)**, or Enet-score **(G, H** and **I)**. *p*-value was calculated by log-rank test.

**Figure 3:**
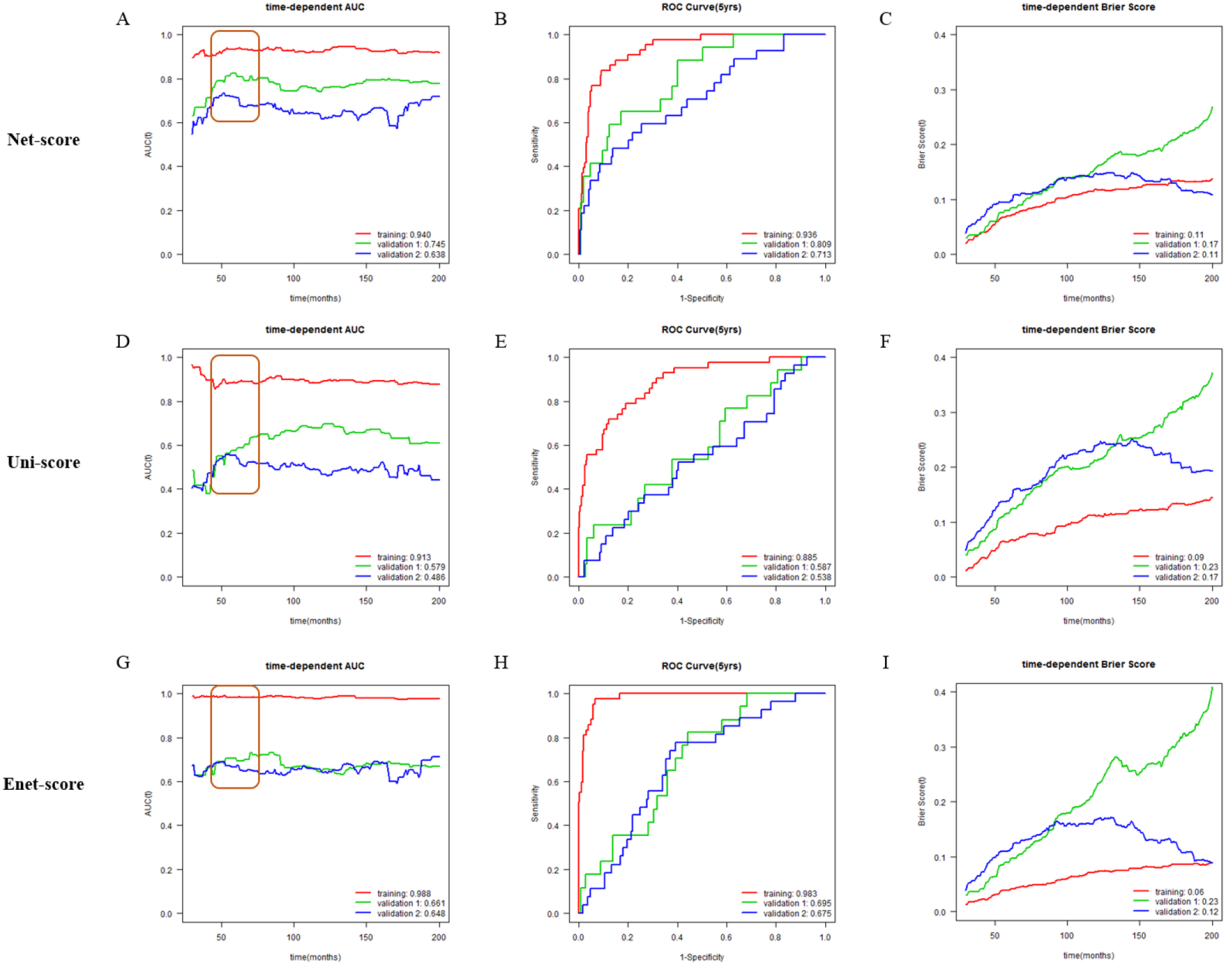
Time-dependent area under the curve (AUC), time-dependent Brier score curve and receiver operating characteristic (ROC) curve at 5 years according to risk scores (Net-, Uni- and Enet-score) in the training and validation sets Time-dependent AUC, time-dependent Brier score curve and ROC curve at 5 years in METABRIC cohort 1 (training, red), METABRIC cohort 2 (validation 1, green), and METABRIC cohort 3 (validation 2, blue) cohorts according to Net-score **(A, B** and **C)**, Uni-score **(D, E** and **F)**, and Enet-score **(G, H** and **I)**. C-indexes are described at the bottom right position of A, D and G. AUC value at 5 years is described at the bottom right position of B, E, and H. Brier scores are described at the bottom right position of C, F and I.

After variable selection and development of prognostic scoring systems, we applied the three systems to the training set (METABRIC cohort 1, n=381) and two validation cohorts (METABRIC cohort 2 and 3, n=167 and 232) to compare their discriminatory accuracy. Using each scoring system, patients of training and validation cohorts were classified into low or high risk groups by the optimum cutoff value of each scoring system. The discriminatory accuracy of the scoring systems was examined with three methods: log-rank test, UNO's C-indexes in time-dependent AUC curve, and AUC values in ROC analysis at 5 years. In the training cohort, all scoring systems could predict patient prognosis (*p*=0 in all score systems) with good statistical significance evaluated by log-rank test. In two validation cohorts, Net-score (*p*=0.000331, 4.58e-06) or Enet-score (*p*=0.0417, 0.000285) showed good statistical significance; however, Uni-score could not discriminate prognosis of patients in the second validation set (*p*=0.0416, 0.318 respectively) (Figure 2). To further compare the accuracy of the three methods, we examined the C-index in time-dependent AUC curve and Brier score for three independent cohorts. Higher C-index and lower Brier score have better prognostic prediction accuracy. In comparison of C-index, Net-score showed better values (0.940, 0.745, and 0.638 in training and two validation cohorts respectively) than the other scores in the validation cohorts; Uni-score and Enet-score showed low values in the two validation cohorts compared to the training cohort (Uni-score: 0.913, 0.579, and 0.485; Enet-score: 0.988, 0.551, and 0.548 in training and two validation sets) (Figure 3A, 3D, 3G and Supplementary Figure 1). In addition, Net-score showed lower Brier score (0.11, 0.17 and 0.11 in training and two validation cohorts respectively) than the other scores in the validation cohorts; Uni-score and Enet-score showed high values in the two validation cohorts compared to the training cohort (Uni-score: 0.09, 0.23, and 0.17; Enet-score: 0.06, 0.23, and 0.12 in training and two validation sets) (Figure 3B, 3E, 3H and Supplementary Figure 1). Notably, Net-score showed smaller differences between AUC curves and Brier scores of three independent cohorts than Uni-score or Enet-score over the entire timeline (Figure 3A, 3C, 3D, 3F, 3G and 3I), suggesting that the overfitting problem was minimum in the Net-based scoring system.

Because cancer-related death usually occurs within 5 years, and 5-year survival rate is commonly used and compared for prognosis prediction of cancer patients, we examined AUC values in ROC analysis and Brier score at 5 years. Net-score showed significantly better values at 5 years in the two validation cohorts (C-indexes: 0.936, 0.809 and 0.713, Brier scores: 0.07, 0.08 and 0.10 in training and two validation cohorts respectively) than the Uni-score (C-indexes: 0.885, 0.587 and 0.538, Brier score: 0.07, 0.12 and 0.14 in training and two validation cohorts respectively) and Enet-score (C-indexes: 0.983, 0.695 and 0.675, Brier scores: 0.04, 0.10 and 0.11 in training and two validation cohorts respectively) (Figure 3B, 3E, 3H and Supplementary Figure 1). Notably, we also observed a smaller standard deviation in AUC values and Brier scores at 5 years between three independent cohorts with Net-score (AUC; 0.11 and Brier score: 0.01) than with Uni-score (0.19 and 0.04) or Enet-score (0.17 and 0.04) (Figure 3B, 3E, 3H and Supplementary Figure 1). Moreover, the standard deviation of C-indexes and Brier scores from Net-score (C-index: 0.15 and Brier score: 0.03) were smaller than those of Uni-score (0.22 and 0.07) or Enet-score (0.19 and 0.09). These results suggest that Uni- and Enet-score models might be suitable for the training cohort but not for the validation cohorts, indicating an overfitting problem.

### Comparison of prognostic score and clinicopathologic variables

**Figure 4:**
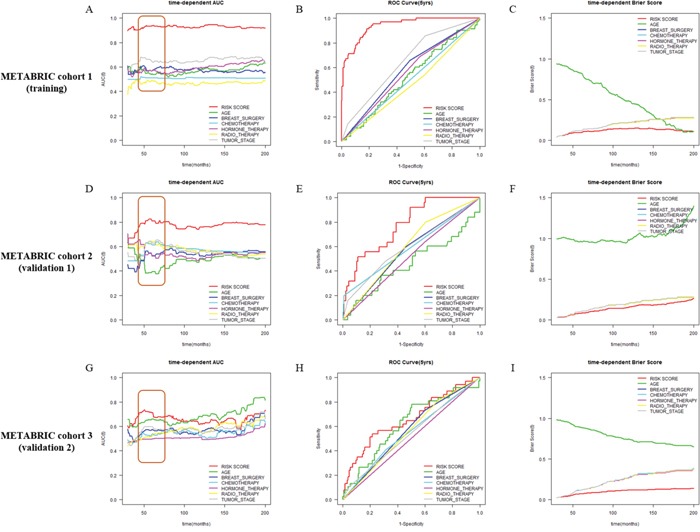
Comparison of discriminative power between Net-score and clinicopathologic variables Time-dependent AUC and ROC curve at 5 years in METABRIC cohort 1 (training, **A**, **B** and **C**), METABRIC cohort 2 (validation 1, **D**, **E** and **F**), and METABRIC cohort 3 (validation 2, **G**, **H** and **I**) cohorts according to Net-score and clinical variables (Net-score: red; age: green; breast surgery: blue; chemotherapy: light blue; hormone therapy: pink; radiotherapy: yellow; tumor stage: gray).

**Figure 6:**
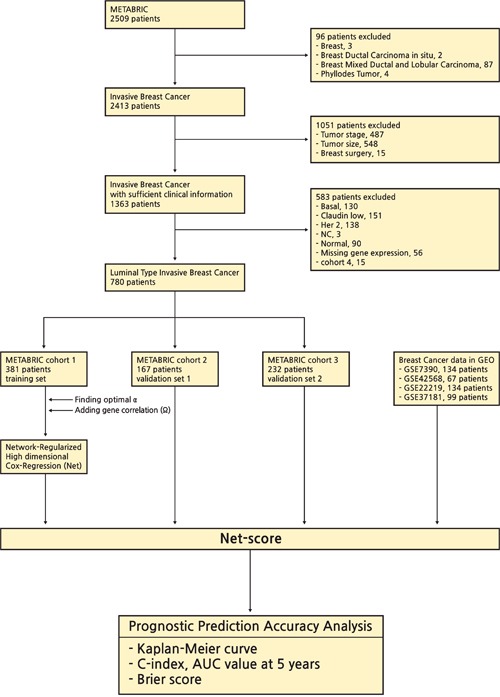
Overview of the study framework

To compare discriminatory power between Net-score and other clinicopathologic variables, we plotted time-dependent AUC, time-dependent Brier score, ROC curve at 5 years (Figure 3 and 4). The Net-score showed significantly higher prognostic accuracy than any other clinical factor including age, surgery type, treatment modality, and tumor stage, especially at 5 years (Figure 3 and 4). Together with Net-score, age also showed good prognostic power in the second validation cohort, which had a wider distribution of age than other cohorts in METABRIC (Supplementary Table 1 and Figure 6A).

### Application to other cohorts (METABRIC and GEO)

**Figure 5:**
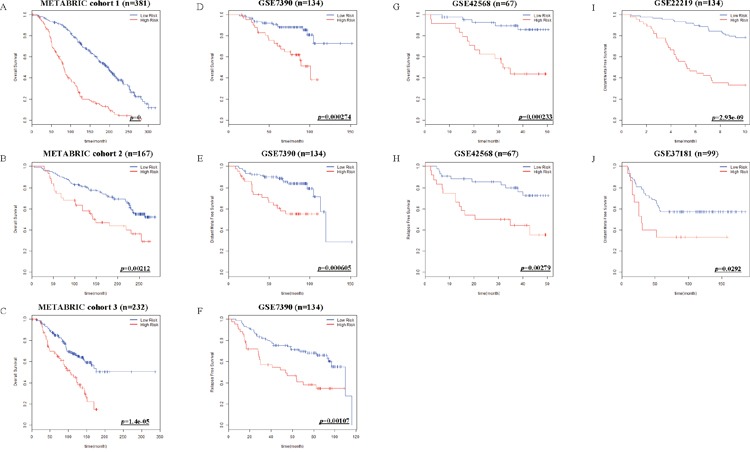
Kaplan-Meier estimates of other survival data of breast cancer patients according to the risk scores Net-score examined overall Survival (OS) in METABRIC cohort 1, 2 and 3 **(A-C)**. OS, Distant Meta Free Survival (DFS) and Relapse Free Survival (RFS) in GSE7390 **(D-F)**, OS and RFS in GSE42568 **(G, H)**, DFS in GSE22219 **(I)**, and DFS in GSE37181 **(J)** were stratified by Net-score. *p* value was calculated by log-rank test.

To confirm the versatility of Net-score, we applied it to various other cohorts. Although we performed variable selection for DSS, the Net-score also could predict overall survival of patients in METABRIC (*p*=0 in training cohort, 0.00212, and 1.42e-05 in validation cohorts Figure 5A-5C). Furthermore, survival data of GSE7390 (*p*=0.000274, 0.000605, and 0.00107 in OS, DFS, and RFS respectively), GSE42568 (*p*=0.000233 and 0.00279 in OS and RFS), GSE22219 (*p*=2.93e-09 in DFS) and GSE37181 (*p*=0.0292 in DFS) data sets are successfully analyzed by Net-score (Figure 5D-5J). The optimum cutoff values are different from value of METABRIC, because their absolute gene expression values are different according to microarray platforms and data processing methods (Table 1).

**Table 1:** Microarray platforms and cutoff values

	Microarray platforms	Cutoff values
	**METABRIC**	
**Cohort 1**	Illumina HumanWG v3.0 platform	3.811765
**Cohort 2**		
**Cohort 3**		
	**GEO databases**	
**GSE7390**	Affymetrix Human Genome U133A platform	8.994179
**GSE42568**	Affymetrix Human Genome U133 Plus 2.0 platform	7.596133
**GSE22219**	Illumina humanRef-8 v1.0 platform	4.256071
**GSE37181**	Illumina HumanWG-6 v3.0 platform	1.51173

### Subgroup analysis

To assess the discriminatory power of Net-score in various subgroups, we classified patients according to mastectomy, menopausal status, and tumor stage. Table 2 summarizes the clinical information of patients in three independent cohorts according to Net-score. Net-score showed good discriminatory power in various subgroups according to surgery type (mastectomy or breast-conserving surgery), menopausal status (pre- or postmenopausal state), or tumor stage (Supplementary Figure 3).

**Table 2:** Clinical characteristics of the training and validation cohorts

Characteristics	High Risk			Low Risk		
Cohort	Training	Validation 1	Validation 2	Training	Validation 1	Validation 2
**No. of patients**	146	44	66	235	123	166
**Age - yrs**	65.14	56.58	65.39	65.55	55.27	63.72
**Breast surgery – no. of patients (%)**						
**Conserving**	60 (41%)	25 (57%)	16 (24%)	109 (46%)	60 (49%)	80 (48%)
**Mastectomy**	86 (59%)	19 (43%)	50 (76%)	126 (54%)	63 (51%)	86 (52%)
**Tumor size (mm)**	30.79	24.70	34.70	23.12	19.20	23.08
**Tumor stage – no. of patients (%)**						
** I**	24 (16%)	22 (50%)	9 (14%)	99 (42%)	89 (72%)	47 (28%)
** II**	105 (72%)	19 (43%)	47 (71%)	130 (55%)	28 (23%)	115 (69%)
** III**	17 (12%)	3 (7%)	10 (15%)	6 (3%)	6 (5%)	4 (3%)
**Menopausal state – no. of patients (%)**						
** Pre-**	12 (8%)	10 (23%)	11 (17%)	28 (12%)	41 (33%)	28 (17%)
** Post-**	134 (92%)	34 (77%)	55 (83%)	207 (88%)	82 (67%)	138 (83%)

## DISCUSSION

For the better prognosis prediction of cancer patients using big genomic data, in the present study, we successfully generated a prognostic scoring system based on Network-regularized high-dimensional Cox-regression (Net). Application of Net for prognosis of breast cancer patients showed superiority over previous methods (stepwise selection via univariate Cox regression and Elastic net). Moreover, we showed its versatile application to other cohorts without overfitting problem commonly observed with previous statistical methods.

Overfitting in a statistical prediction system, characterized by high accuracy for a classifier when evaluated on the training cohort but low accuracy when evaluated in independent validation cohorts, has been reported as a serious problem in high-dimensional data [5]. Stepwise selection via univariate Cox regression model is the most commonly used method for developing a prognostic gene signature. However, this approach has a serious overfitting problem because it only considers individual effects of variables (Figure 2D-2F and Figure 3G-3I). Similarly, Elastic net could not overcome the overfitting problem (Figure 2G-2I; Figure 3G-3I). Comparison of prediction accuracy analysis showed that Uni-score and Enet-score had larger differences between training and validation cohorts than Net-score (Figure 3). Therefore, these previous methods may not be as suitable for selection of a subset that can predict patient survival. Because we need more information on variables in order to reduce overfitting, we made a gene correlation matrix by combining six large pathway databases. We finally developed a Net-based scoring system that can predict prognosis using a new gene correlation matrix (Figure 1B). In addition, the discriminatory power was higher than that of previous methods, whereas the standard deviation was smaller. These results suggest that a Net-based scoring system has minimal overfitting and high discriminatory power compared with previous statistical methods.

Many prognosis prediction systems for the survival of cancer patients that are based on clinical data for gene expression have been developed: 21 genes and 70 genes for breast cancer, 5 genes for hepatocellular carcinoma, 5 genes for non-small cell lung cancer [15–21]. However, their usefulness in the treatment of cancer patients might be limited because selection of gene sets for the prediction system was not systemic or statistical and the gene sets were tested using univariate regression. Although great results have been obtained using univariate regression, these systems might suffer from the overfitting problem because they did not consider the importance of the grouping effect. As shown in Figure 5, Net-score is very useful to predict other breast cancer data sets because it considers gene-gene interaction with other grouping effects.

Breast cancer is the most common malignancy in women in developed countries, with luminal type representing approximately 60–70% of all cases [22]. Luminal breast cancer has varied clinical outcomes despite similar molecular patterns, making it difficult to predict prognosis [22]. The results of our advanced technique were highly associated with various survival data and had high reproducibility. This method could be useful in stratifying patients according to risk of luminal breast cancer and helpful for deciding treatment options.

Clinical and genetic information included in METABRIC is uniform and well-designed [8, 9] even though the cohorts in METABRIC were collected independently. For these reasons, three cohorts in METABRIC were the best to develop and validate prognostic system. At the process of variable selection, almost all clinical variables such as age, stage, Nottingham prognostic index (NPI), therapeutic modalities and tumor size, were examined. Among clinical variables, tumor size was always selected at the variable selection. Although some clinical or genetic variables in data set were previously known to be important for prognosis, they cannot be selected due to multicollinearity problem. If the variables in Net-score are highly correlated with each other, it will cause multicollinearity problem. For example, Nottingham prognostic index (NPI), which includes tumor size, lymph node status and histological grade information, and tumor stage are well-known prognostic factor for breast cancer, however, they were not selected in the current study [23, 24]. NPI or tumor stage has high correlation coefficients with tumor size (Supplementary Figure 2, NPI-tumor size: 0.55, tumor stage-tumor size: 0.48 calculated by spearman correlation or Point-Biserial correlation respectively). As another example, because KIF20A in Net-score has high correlation with many other well-known prognostic genes in oncotype DX and MammaPrint, KIF20A may represent their prognostic effect (Supplementary Figure 4) [17, 21]. Although The Cancer Genome Atlas (TCGA) is an excellent database providing information about the expressional status of mRNA, it does not provide information about the tumor size, which is the reason TCGA was not included for the present analysis.

The advantages of the Net-based scoring system are as follows: (1) the variable selection method considers biological network information from six large pathway databases. (2) It can predict other cancer cohorts well because it has the least overfitting with high discriminatory power compared with other commonly used statistical methods. In conclusion, we believe that Net scoring system is better than any other previous statistical methods for prognostic prediction in cancer patients.

## MATERIALS AND METHODS

### Patients

The breast cancer data set was released by the METABRIC study (Illumina Human WG v3, n=780) [8, 9]. The mRNA expression and clinical data were downloaded from cBio Cancer Genomics Portal. This process was performed by using ‘*cgdsr*’ package in R. The criteria for inclusion were as follows: (1) patients with invasive breast cancer (n=96); (2) patients with insufficient information about tumor stage (n=1036), and surgery type (n=15); (3) patients with non-luminal type breast cancers (Basal, n=130; Claudin-low, n=151; Her 2, n=138; Normal, n=90; NC, n=3); (4) patients with unavailable gene expression values (NA, n=56). Although 15 patients in cohort 4 have all clinical and genetic data, we could not help excluding them due to small number of patients. All samples were obtained with consent from the patients and appropriate approval from ethical committees. We chose the cohort with the largest number of patients (n=381) as a training cohort and analyzed the two other large cohorts as validation sets (n=167, 232). The flowchart of this study are described in Figure 6. Clinical characteristics of patients in training and validation cohorts are summarized in Table 2.

Other cohorts were collected from the Gene Expression Omnibus (GEO). We searched all public data sets and then we excluded GEO data that did not have clinical data (survival and hormone receptor data) and variables in Net-score. In each data sets, we only included patients with hormone receptor positive because luminal type breast cancers are hormone positive cancers. The mRNA expression data with patient's information are available on GEO ( https://www.ncbi.nlm.nih.gov/geo), with accession numbers are GSE7390 (Affymetrix Human Genome U133A Array, n=134), GSE42568 (Affymetrix Human Genome U133 Plus 2.0 Array, n=67), GSE22219 (Illumina humanRef-8 v1, n=134) and GSE37181 (Illumina Human WG-6 v3, n=99) [10–14]. The expression and clinical data from GEO were downloaded by using ‘*GEOquery*’ package in R.

### Network-regularized high-dimensional Cox regression (Net)

To obtain more significant results using Net, additional information about gene–gene correlation is required. We converted pathway topology information from six large databases (Biocarta, HumanCyc, KEGG, NCI, Panther, and Reactome) to the gene network using the R package ‘*graphite*’. This package provides gene–gene interaction information in the form of edges that represent direct or indirect interactions. We made a gene correlation matrix from the combined six databases. This provided a greater grouping effect because it had more information on gene–gene interaction. We performed Net implemented in the R package ‘*coxnet* (version 0.2)’ to evaluate the association between disease-specific survival (DSS) and various subsets considering mRNA expressions of genes and clinical variables (age, tumor stage, tumor size, Nottingham prognostic index (NPI), therapeutic modalities, hormonal receptor status and grade) together using the ‘leave-one-out’ method for cross-validation. mRNA expression values, age, tumor size and NPI were numeric continuous values and other clinical variables were categorical factors. Categorical factors were transformed to dummy variables for Cox regression. The mixing parameter α, which decides the balance between Ridge and Lasso penalties, with minimal Cross-Validation (CV) error was determined.

### Stepwise selection via univariate Cox regression

Variables were prepared as described above in the ‘Network-regularized high dimensional Cox regression (Net)’ section. We performed univariate Cox proportional hazard regression to evaluate the association between DSS and variables including genes and clinical factors in the training cohort. To avoid the curse of dimensionality, we used stepwise variable selection for the top 100 genes/factors that were highly correlated with survival.

### Elastic net

Variables were prepared as described above in the ‘Network-regularized high dimensional Cox regression (Net)’ section. We used Elastic net implemented in the R package ‘*glmnet* (version 2.0.5)’ to evaluate the association between DSS and variable subsets considering both genes and clinical variables using the “leave-one-out” method for cross-validation. The mixing parameter α, which decides the balance between Ridge and Lasso penalties, with minimal CV error was determined.

### Prognostic scoring system

We used a linear combination of the values of variables by regression coefficients to calculate a prognostic score (Supplementary Table 1). The regression coefficients from training set were also used to make prognostic score in other data sets. In each analysis we determined the optimum cutoff that had the maximal UNO's C-index [25], which represents the discriminatory power average by 5-fold cross-validation. To know the differences of variable values in risk features, we compared the variable values from three models in METABRIC cohorts (Supplementary Table 2).

### Application to other survival data

We performed survival analysis of breast cancer patients in other survival data by using Net prognostic score. In METABRIC data set, there are two kinds of survival data. One is DSS and the other is overall survival (OS). The prognostic score from DSS was also applied to METABRIC (OS) and GSE7390 (OS, Distant Meta Free Survival (DFS), Relapse Free survival (RFS)), GSE42568 (OS and RFS), GSE22219 (DFS) and GSE37181 (DFS).

### Correlation analysis

Spearman's correlation coefficient was used to analyze two continuous variables. The correlation between categorical variable (tumor stage) and continuous variable (tumor size) was analyzed by Point-Biserial correlation coefficient.

### Discriminatory accuracy analysis

To evaluate the discriminatory accuracy of statistical methods, we used three methods: log-rank test; UNO's C-index in the time-dependent area under the curve (AUC) analysis; and AUC value in receiver operating characteristics (ROC) analysis at 5 years. Log-rank test, UNO's C-index, and AUC value were obtained using R package ‘*survival*’ and ‘*survAUC*’. ROC curve analysis showed discriminatory power of variables including prognostic gene sets and clinical factors. We calculated AUC values in ROC analysis that ranged from 0.5 to 1.0 using R package ‘*survAUC*’. The value of AUC means were as follows:

**Table:** 

	0.5	No discrimination
	0.6 ~ 0.7	Poor
AUC value	0.7 ~ 0.8	Acceptable (fair)
	0.8 ~ 0.9	Excellent (good)
	0.9 ~ 1.0	Outstanding

The UNO's C-index provides a global assessment of a fitted survival model for the continuous event time rather than focusing on the prediction of t-year survival for a fixed time. We used R software version 3.3.0 for all analyses.

## SUPPLEMENTARY MATERIALS FIGURES AND TABLES







## Abbreviations

METABRIC: Molecular Taxonomy of Breast Cancer International Consortium
GEO: Gene Expression Omnibus
Net: Network-Regularized high-dimensional Cox-regression
Enet: Elastic net
TCGA: The Cancer Genome Atlas
ICGC: International Cancer Genome Consortium
DSS: Disease-specific survival
NPI: Nottingham prognostic index
CV: Cross-Validation
OS: Overall survival
DFS: Distant Meta Free Survival
RFS: Relapse Free survival
AUC: Area under the curve
ROC: Receiver operating characteristics
