# Supplementary material for: Gene network inherent in genomic big data improves the accuracy of prognostic prediction for cancer patients
Source: Oncotarget. 2017 Aug 24;8(44):77515–26. doi: 10.18632/oncotarget.20548 (PMC5652797; doi:10.18632/oncotarget.20548)
Supplement: Supplementary file 1 [file oncotarget-08-77515-s001.pdf]

Gene network inherent in genomic big data improves the accuracy of prognostic prediction for cancer patients

SUPPLEMENTARY MATERIALS

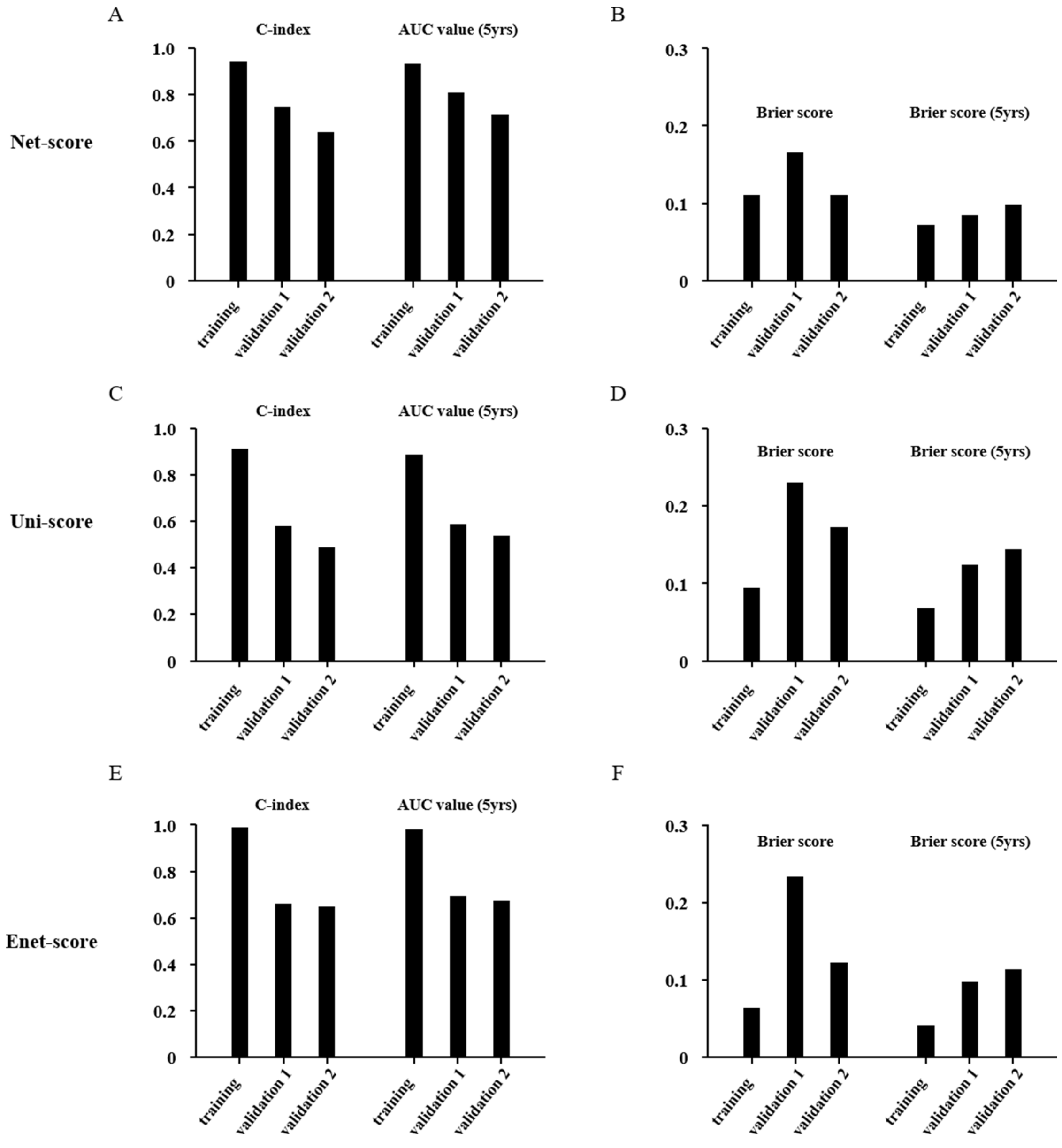

Supplementary Figure 1: Histograms of prediction accuracy values according to the risk scores (Net-score, Uni-score and Enet-score). Histograms of C-indexes and AUC values at 5 years according to risk scores (Net-score: **A**, Uni-score: **C** and Enet-score: **E**). Histograms of Brier scores and Brier scores at 5 years according to risk scores (Net-score: **B**, Uni-score: **D** and Enet-score: **F**).

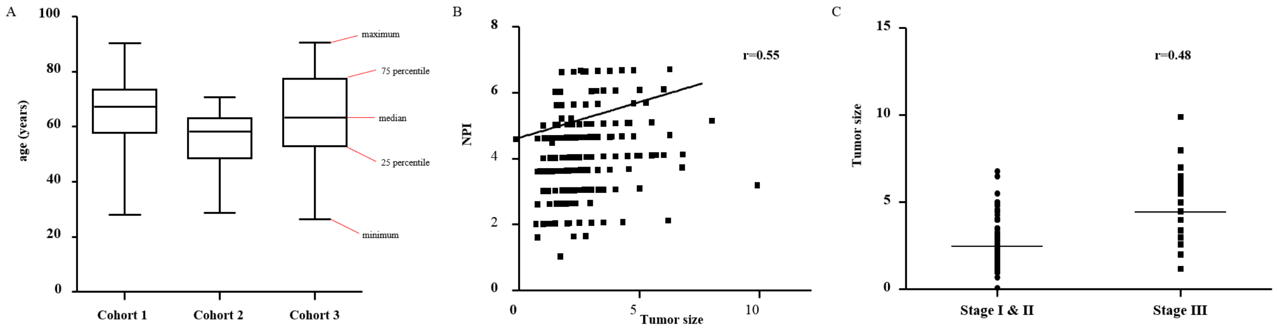

Supplementary Figure 2: **(A)** Box plot of age distribution in training and two validation cohorts. **(B)** Dot plot for correlation between Nottingham Prognostic Index (NPI) and tumor size. The correlation coefficient was calculated by Spearman's rank correlation method (r=0.55) **(C)** Box plot for correlation between tumor size and Stage (I & II and III). The correlation coefficient was calculated by Point-Biserial correlation method (r=0.48).

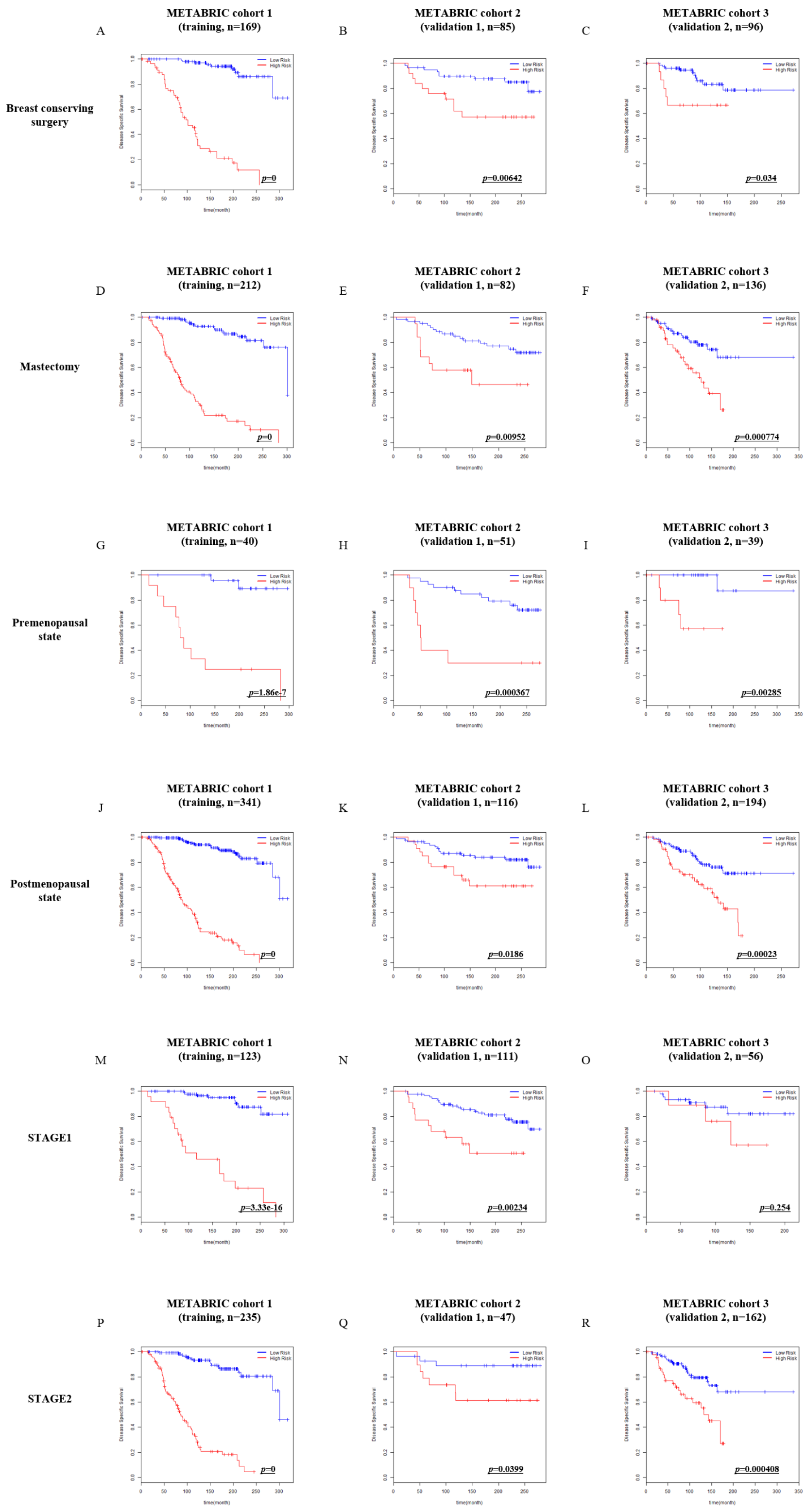

Supplementary Figure 3: Evaluation of Net-score in various subgroups of breast cancer patients. Disease-specific survival (DSS) according to Net score for various subgroups (panels **A-C**: Breast-conserving surgery, panels **D-F**: Mastectomy, panels **G-I**: Premenopausal state, panels **J-L**: postmenopausal state, panels **M-O**: Stage 1, and panels **P-R**: Stage 2) for METABRIC cohort 1 (training), METABRIC cohort 2 (validation 1), and METABRIC cohort 3 (validation 2) respectively. *p*-value was calculated by log-rank test.

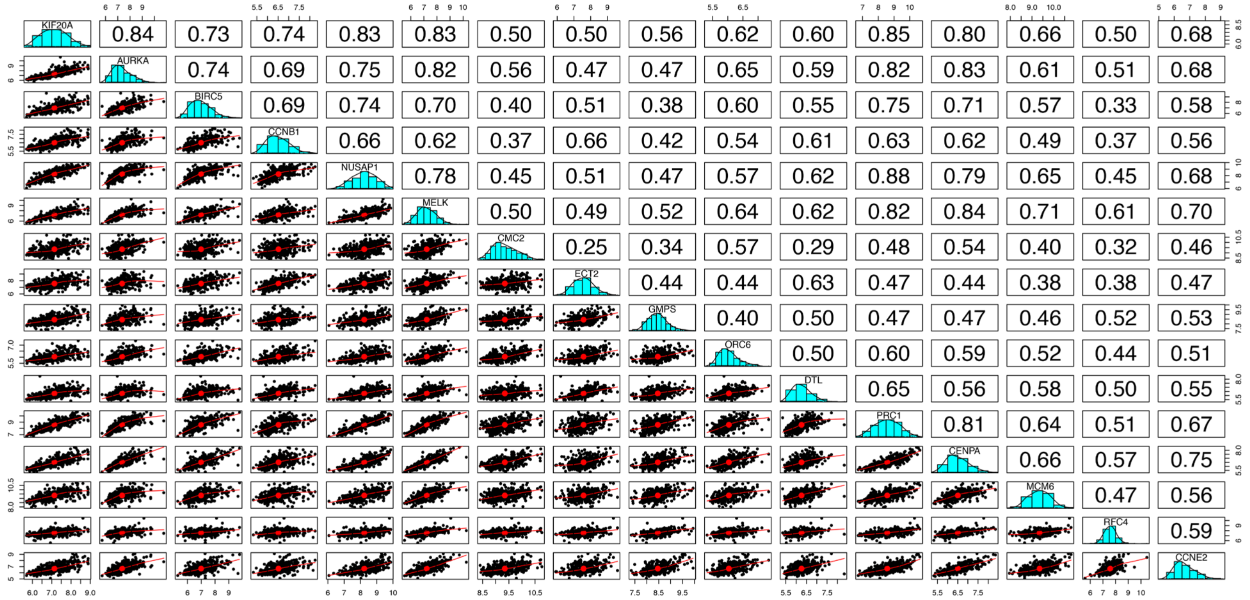

Supplementary Figure 4: Scatterplot for gene-gene correlation. The plot has histogram of the each gene expressions in the diagonal, scatterplot in the lower part of the matrix, and spearman correlation coefficients in the upper part of the matrix.

Supplementary Table 1: Estimated regression coefficients via Network-regularized high-dimensional Cox-regression (Net), Univariate Cox-regression and Elastic net

See Supplementary File 1

Supplementary Table 2: The differences of variables from three models between high and low risk groups

See Supplementary File 2
